# Acute Coronary Syndrome in a 40-Year-Old Male With Coronary Ectasia: A Case Report

**DOI:** 10.7759/cureus.75213

**Published:** 2024-12-06

**Authors:** Krupal C Reddy, Waleed Kadro, Rafael Moguel Ancheita, Rajani Prakash

**Affiliations:** 1 Zulekha Hospital, Interventional Cardiology, Dubai, ARE; 2 Zulekha Hospital, Interventional Cardiology, Sharjah, ARE; 3 The Clinics of the Heart/Costamed, Interventional Cardiology, Cozumel, MEX; 4 Zulekha Hospital, Intensive Care Unit, Sharjah, ARE

**Keywords:** acute coronary syndrome, coronary ectasia, left anterior descending artery, lumen dilation

## Abstract

Coronary artery ectasia (CAE) is a relatively uncommon condition that is often identified incidentally during coronary angiography. It can lead to altered hemodynamics and an increased risk of thrombotic events. This case report outlines the clinical progression of a 40-year-old male diagnosed with acute coronary syndrome (ACS). Coronary angiography demonstrated complete occlusion of the ectatic left circumflex artery (LCx) and dominant right coronary artery. The patient underwent thrombus aspiration, which reinstated normal coronary flow (TIMI III) and uncovered a large eccentric, ball-like thrombus within the vessel. Stenting was not feasible due to the size of the ectasia. This case illustrates the diagnostic and therapeutic challenges in managing ACS in patients with CAE and emphasizes the need for additional research to determine optimal treatment strategies for enhancing long-term outcomes.

## Introduction

Coronary artery ectasia (CAE) refers to coronary artery lumen dilation that exceeds more than one-third of coronary artery length and has 1.5 times more dilated segment diameter than the normal adjacent segment diameter [1]. The incidence of CAE varies between 0.3 and 4.9% [2]; its exact pathophysiology remains unknown, often classified as an anatomical variant or clinical variation of the coronary syndrome [1]. It has a prevalence rate of 1.2-4.9%, with a male-to-female ratio of 3:1 [2]. Some of the risk factors for CAE are smoking, hypertension, and use of drugs like cocaine. Coronary angiography, either catheter- or non-invasive CT or MRI, can sometimes be useful when other methods like CT or invasive angiography are contraindicated. Also, intravascular ultrasound (IVUS) may help characterize the luminal and particular pathologies [3,4]. The management of CAE poses significant challenges due to its rarity and the scarcity of large randomized trials providing insights into treatment strategies, including infrequent surgical interventions. Additionally, CAE presents a multifaceted challenge for clinical and interventional cardiologists, as each therapeutic approach offers unique advantages [1,5].

## Case presentation

**Figure 1:**
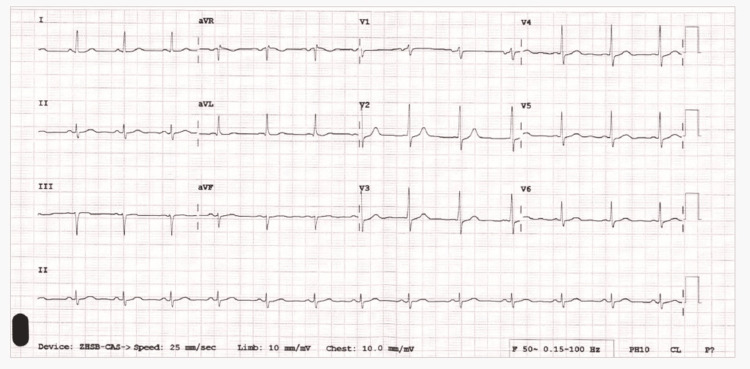
Initial electrocardiogram in the emergency room The initial electrocardiogram shows a heart rate of 78 beats per minute along with T-wave inversion in leads II, III, and AVF

The patient was a 40-year-old male who presented to the emergency room complaining of central chest pain radiating to the back and left arm and dyspnea for the past hour; he did not have any cold, cough, or a history of fever. The patient was a non-smoker and had never smoked in the past. His past medical history included hypertension, dyslipidemia, supraventricular tachycardia on medication, and mild intermittent asthma managed with a salbutamol inhaler as needed. The baseline lipid levels were as follows: low-density lipoprotein (LDL): 102.3 mg/dl; total cholesterol: 153 mg/dl; high-density lipoprotein (HDL): 31.3 mg/dl; and triglycerides: 130 mg/dl. On examination, he had a heart rate of 68 beats per minute, BP of 100/60 mmHg, respiratory rate of 16/minute, and 98% oxygen saturation on room air. The patient had significantly elevated serial troponin I levels, starting at 4.3 ng/mL, then rising to 45 ng/mL, and eventually reaching 2100 ng/mL, indicating ongoing myocardial injury. The electrocardiogram showed a biphasic T wave in lead II with left axis deviation (Figure 1).

**Figure 2:**
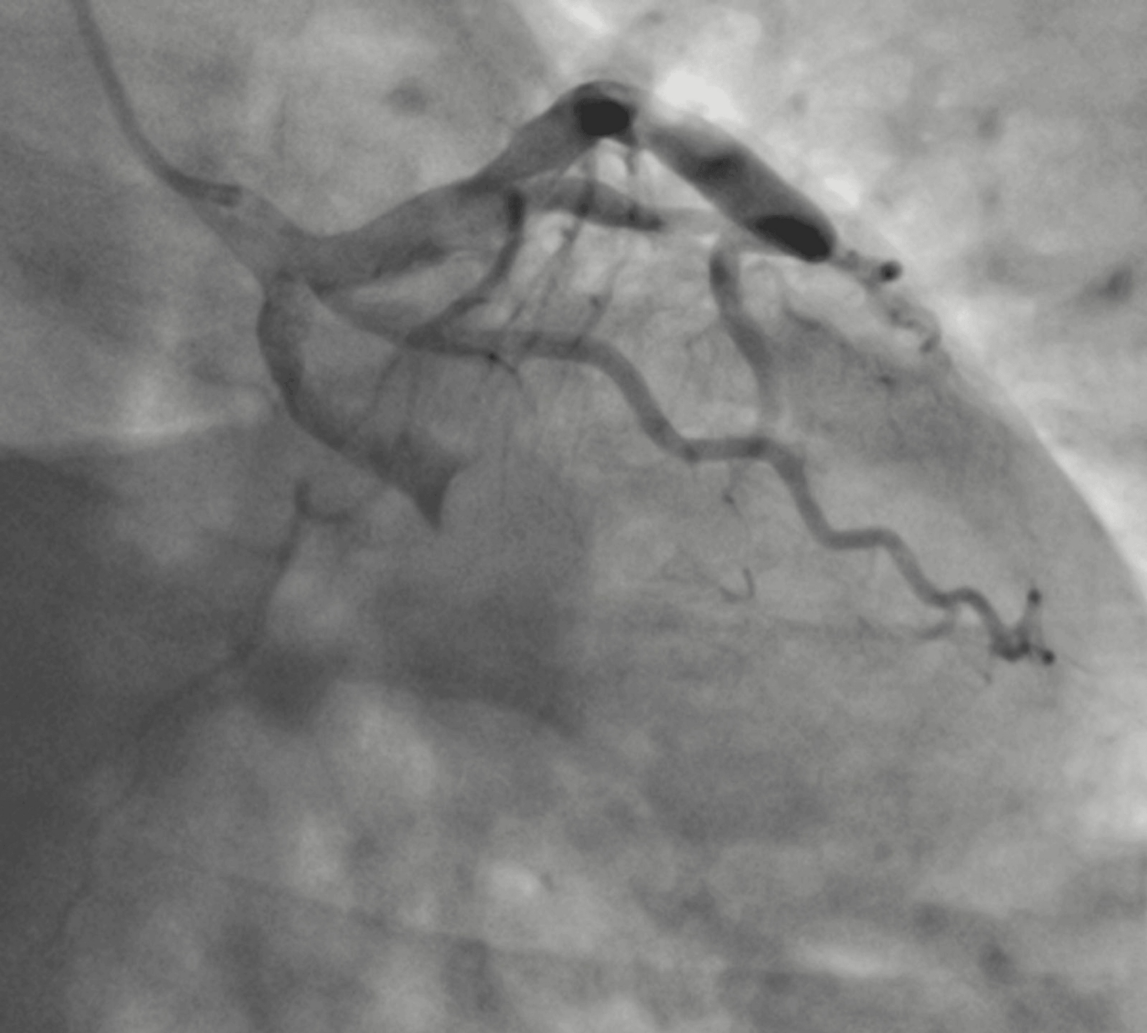
Coronary angiography The angiographic image shows the presence of coronary artery ectasia

**Figure 3:**
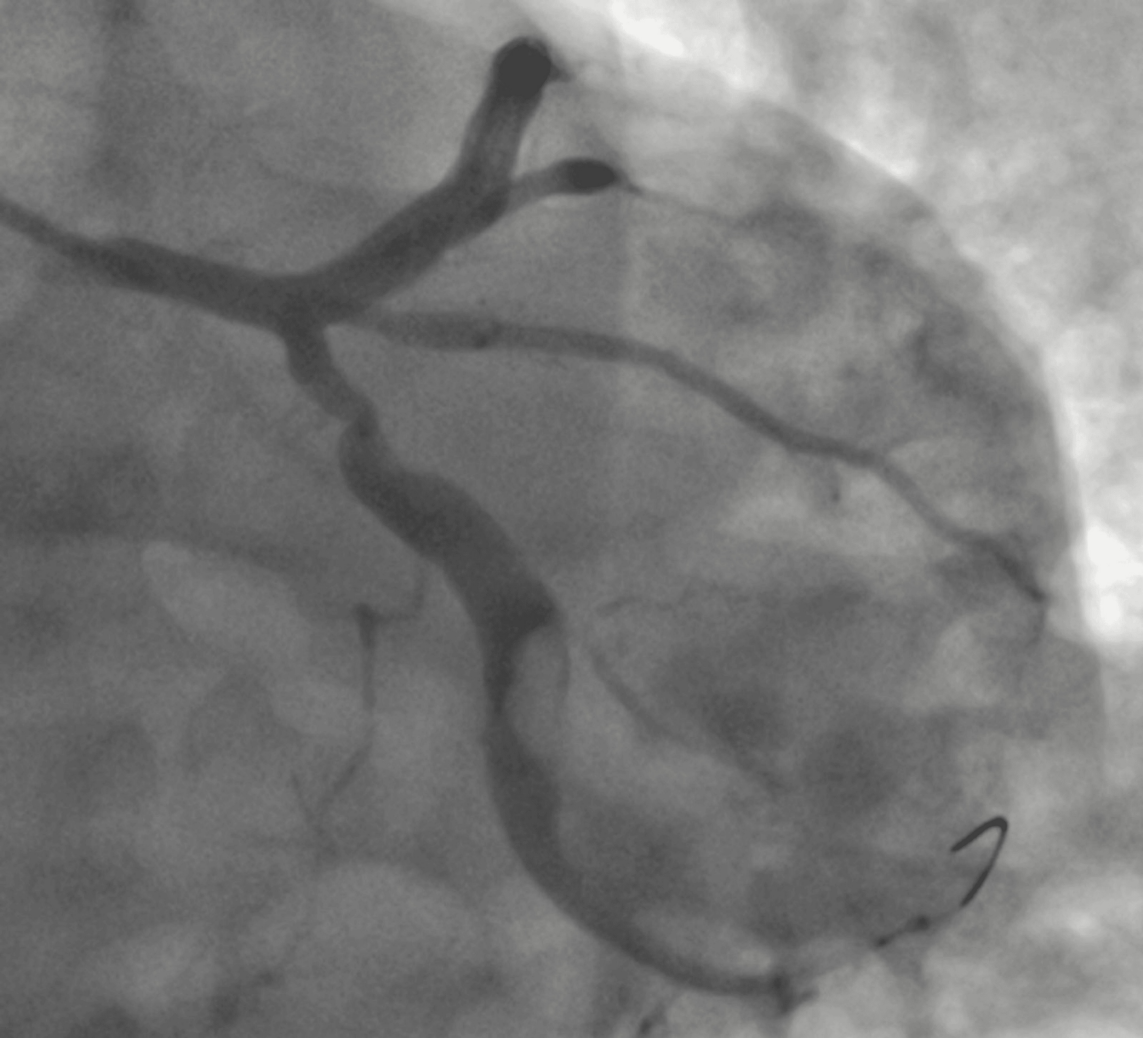
Coronary angiography indicating coronary artery ectasia with thrombus

The transthoracic echocardiography revealed inferior wall hypokinesia, 40% left ventricle ejection fraction, and mild mitral regurgitation. The coronary angiography (Figure 2) revealed a complete occlusion of the culprit vessel and the left circumflex artery (LCx), with a large eccentric thrombus and ectasia identified after aspiration (Figure 3) during the percutaneous intervention. The guidewire successfully crossed into the Cx, followed by thrombus aspiration using a thrombuster catheter (Kaneka Medix, Minato, Japan), retrieving large clots after several attempts, and restoring TIMI-III flow. However, a ball-like eccentric thrombus, approximately 10 mm in diameter, remained. Given the significant ectasia, any further instrumentation, such as IVUS, was not performed to avoid rare but possible complications like thrombus migration or vessel wall injury.

The prescription at discharge included ramipril 5 mg once daily to control blood pressure, aspirin 100 mg once daily as an antiplatelet agent, atorvastatin/ezetimibe 40/10 mg once daily for lipid management, and bisoprolol 5 mg once daily to manage heart rate and arrhythmia prevention. The patient was advised to have regular clinical follow-ups, as well as to adopt lifestyle and diet modifications. Anticoagulation therapy was initially started with rivaroxaban 15 mg twice daily, then shifted to 15 mg once daily after one month. At six months, the patient's ECHO revealed normal left ventricular systolic function without regional wall motion abnormality and even his stress test was negative for inducible ischemia. After one year, the rivaroxaban dose was reduced to 2.5 mg twice daily as a maintenance dose, although no specific guidelines exist regarding the dosage of anticoagulation in patients with coronary ectasia.

## Discussion

CAE is a rare finding identified during coronary angiography. The right coronary artery is the most frequently observed site (68% of cases), followed by the proximal left anterior descending artery (60%) and LCx (50%) [6]. In a study involving both children and adults, Kawasaki disease was found to be the most common cause [7]. Atherosclerosis was responsible for over 50% of adult cases [8], due to the dilation of the vessel lumen after vascular remodeling, which compensates for the growth of atherosclerotic plaques. CAE can be associated with apical hypertrophic cardiomyopathy, where the high tension in the wall leads to its formation. Also, interventions related to post-percutaneous coronary, like balloon angioplasty, stent placement, and atherectomy, can lead to aneurysm formation due to iatrogenic injury to the blood vessel media [9].

Doi et al. have reported that coronary abnormalities significantly increased the risk of major adverse cardiac events by 3.25 times, that of cardiac death by 2.71 times, and nonfatal myocardial infarctions by 4.92 times. They also observed that over 70% of CAE patients experienced ischemia in nonobstructive coronary segments after surviving a myocardial infarction. This may be due to slow blood flow in dilated vessels that leads to an increase in blood viscosity and coagulation; the authors observed, over 49 months, 33% fewer major adverse cardiac events incidence in their eight patients [10]. Coronary angiography is the gold-standard procedure for diagnosing CAE. Angiographic imaging can provide indications of both laminar and turbulent blood flow. The dye may accumulate in enlarged sections of the coronary arteries, necessitating additional time for complete filling.

CAE is closely related to coronary artery disease. In a study by Liu et al., 99 patients with CAE underwent baseline coronary angiogram followed by a second angiogram as late as 16 years later. The researchers observed that all patients initially had atherosclerosis, which had worsened significantly over time, and the extent of ectasia showed minimal change. The study suggests that preventing atherosclerosis progression may be more clinically important than treating ectasia [11]. The management of CAE is similar to that of coronary artery disease, including stent angioplasty and surgery. Although there are no randomized studies specifically evaluating medical therapy for CAE, aspirin is advised for all patients due to the condition's frequent association with obstructive coronary lesions [12].

Gunasekaran et al. investigated the long-term effects of dual antiplatelet therapy and anticoagulation in patients affected by CAE over 9.7 years of mean follow-up. Among 121 patients treated with dual antiplatelet therapy, the ACS incidence was significantly lower compared to 91 patients who did not receive dual antiplatelet therapy (17% and 34%). Similarly, in 105 patients receiving systemic anticoagulation, the incidence of ACS decreased compared to 91 patients who did not receive it (29% and 42%); this illustrates the benefit of these treatments [13,14].

## Conclusions

This report highlights the challenges faced in the clinical management of ACS in patients with CAE, a complex combination that warrants immediate diagnosis and therapeutic strategies. In this case, the approach involved combining anticoagulants, antiplatelet agents, and lipid-lowering therapy. CAE, although rare, may present with adverse cardiac events that need regular follow-ups and efficient risk factor management. This report underlines the need for further research into the pathophysiology and optimal treatment strategies for CAE, including close long-term follow-up and prevention of recurrent ischemic events.
